# Insomnia and Inflammation Conspire to Heighten Depression Risk: Implications for Treatment and Prevention of Mood Disorders

**DOI:** 10.1016/j.biopsych.2025.04.018

**Published:** 2025-05-04

**Authors:** Michael R. Irwin

**Affiliations:** Cousins Center for Psychoneuroimmunology, Jane and Terry Semel Institute for Neuroscience and Human Behavior, University of California, Los Angeles, California; and the Department of Psychiatry and Bio-behavioral Sciences, David Geffen School of Medicine, University of California, Los Angeles, California.

## Abstract

Insomnia is ubiquitous, is comorbid with all major mental disorders, increases the risk of depression, and contributes to inflammatory morbidity and all-cause mortality. This review examines the relationships between insomnia and inflammation in the pathophysiology of depression. The unique role of insomnia on depression risk is examined with interrogation of what aspects of sleep disturbance contribute to depressed mood. Furthermore, the influence of insomnia and its specific aspects (i.e., short sleep duration, disturbance of sleep maintenance) on affective mechanisms are considered, with a focus on reward activation and emotion processing. Given that inflammation contributes to some types of depression, the bidirectional interactions between sleep and inflammation are examined with consideration of how sleep deprivation induces activation of systemic, cellular, and genomic inflammatory outcomes and the causal role of inflammation in precipitating depressed mood and depressive symptoms. Key gaps in the literature linking insomnia and inflammation to depression risk are identified, and maps for future research are proposed. In particular, this review considers how the components of insomnia and inflammation conspire together to exaggerate deficits in reward activation and recognition of emotion, which underlie depression risk and adverse depression outcomes. Finally, informed by this two-hit model of insomnia and inflammation on depression risk, this review examines the efficacy of behavioral interventions that target insomnia and reverse related inflammation and discusses their potential to refine therapeutic approaches for depression treatment and prevention in individuals with insomnia.

Occasional sleep disturbance including difficulty falling sleep, wake after sleep onset (WASO), or nonrestorative sleep is ubiquitous. Unfortunately, these complaints persist over days and weeks for many people, with one-third of adults (1) and more than two-thirds of older adults reporting at least 1 persistent insomnia complaint (2,3). Nearly 1 in 10 adults fulfill the DSM-5 criteria for insomnia disorder, which is characterized by these sleep complaints, their frequency (i.e., 3 nights per week), and a 3-month duration (2). Furthermore, insomnia is often comorbid with all major mental disorders, increases the risk of depression and inflammatory disorders, and contributes to all-cause mortality (3). Hence, there is a pressing need to understand the affective and biological mechanisms that might be targeted to mitigate the morbidity associated with insomnia. In this review, the relationships between insomnia and depression will be examined, taking into account the influence of insomnia and its specific components on affective mechanisms of depression risk including reward and emotion perception processes. Additionally, given that inflammation is thought to contribute to some types of depression and that insomnia, including disturbance of sleep maintenance and short sleep duration, induces the activation of inflammatory biological mechanisms, the separate and joint contributions of insomnia and inflammation on depressive symptoms, reward activation, and emotion perception will be discussed. Finally, to inform the clinical implications of these mechanisms for improving depression outcomes, this review will consider the potential of behavioral interventions that target insomnia and reverse inflammation to treat and prevent depression.

## INSOMNIA AND DEPRESSION RISK

During an episode of depression, insomnia symptoms are found in nearly all patients (4), occurring as a prodromal symptom and often persisting after affective symptoms remit. Moreover, insomnia disorder, as well as insomnia complaints, predicts depression, contributing to a 2-fold greater likelihood of developing a major depressive disorder (5–9) and also increasing the risk of suicide (10). Furthermore, this prospective association remains robust even after adjusting for baseline depressive symptoms, depression history, or antidepressant use, all of which are independently associated with depression (7,10,11).

Duration of insomnia, medical comorbidity, and various insomnia characteristics further contribute to an increased risk of depression. For example, when insomnia persists over months, the risk of depression substantially increases (12), with evidence that persistent insomnia over 1 year is associated with a 14-fold increase in the likelihood of depression over the subsequent year (13). Furthermore, depression risk is elevated in individuals who have comorbid inflammation-related medical conditions, and insomnia disorder further escalates this risk (14). For example, among HIV1 men who have sex with men, insomnia complaints are prospectively associated with >30% annual incidence of depression; the global annual incidence of depression is estimated at 5% (15). One aspect of insomnia is disturbance of sleep maintenance, and when indexed by actigraphy assessment of WASO, disturbance of sleep maintenance shows a unique association with depressive symptoms in older adult females (16). Finally, experimental sleep deprivation (i.e., short sleep duration) induces increases in depressed mood, with exaggerated response in older adults and individuals with an inflammatory disorder (14,17).

These observational data have important clinical implications for the treatment and prevention of depression. Indeed, among the salient risk factors associated with depression in older adults including comorbidity, disability, prior depression, and female sex, insomnia is the only modifiable risk factor (18). Moreover, understanding the affective and biological mechanisms by which insomnia is converted to depression is needed to inform the development of personalized approaches in the treatment and prevention of depression.

## INSOMNIA AND AFFECTIVE MECHANISMS OF DEPRESSION RISK

Affective mechanisms, such as reward processes and facial emotion perception (FEP), might serve as pathways linking insomnia and depression. As such, evaluating the impact of insomnia on these mechanisms serves as an important step toward advancing strategies for the development of interventions that target these modifiable processes and mitigate depression risk.

### Reward Processes

Anhedonia or loss of pleasure is a key symptom of major depression, which is characterized by reduced wanting for pleasure (i.e., reduced reward motivation) or reduced liking of pleasure (i.e., reward sensitivity). Deficits in reward motivation or reward sensitivity commonly occur in depression (19), are found in nondepressed populations at familial risk for depression (20,21), and prospectively correlate with incident depression and onset of subthreshold depressive symptoms (22,23). Notably, older adults show an increased vulnerability to anhedonia in the context of depression (24), possibly due to age-related decreases in frontostriatal dopaminergic function (25).

Limited research has examined the link between insomnia and reward processes. Insomnia disorder is associated with alterations in neural substrates linked to reward pathways including reduced gray matter volume and functional connectivity in reward-related brain regions (26), and daily sampling of insomnia complaints correlated with decreases in wanting and liking reward experiences in adults (27). In older adults with insomnia disorder, Boyle *et al*. (28) recently found reduced reward motivation. Those with insomnia were less likely to select hard trials (e.g., high effort and high reward), and the severity of insomnia symptoms correlated with fewer choices of hard trials (28). Although females, compared with males, showed reduced reward motivation, males with insomnia were most likely to have reward deficits. Similar findings were found for reward sensitivity (28).

Sleep deprivation, in contrast to insomnia disorder, induces a state of labile affective imbalance with overresponding to both negative and positive cues (29). For example, similar to the increases in negative affective responding in individuals with insomnia disorder, sleep deprivation induces amplification of amygdala reactivity in response to negative emotional stimuli (30). Yet, in contrast to insomnia, sleep deprivation leads to increases, not decreases, in the activity of mesolimbic brain networks involved in reward processing of positive emotional experiences (i.e., bias in labeling visual stimuli as being pleasant) (29), greater increases in ventral striatal activity task in response to monetary reward tasks (31), and greater activation in the right nucleus accumbens in response to a gambling task (32).

### Emotion Perception

Another affective mechanism influenced by insomnia is FEP, which is defined as the sensory ability to evaluate emotional information from facial expression (33,34). Facial emotion tasks query both emotion recognition and perceived emotion intensity. In patients with depression, brain regions involved in the processing of emotions are altered (35,36), which leads to impaired emotion recognition, especially recognition of sad facial expressions (37–41). Furthermore, the severity of depressive symptoms correlates with less accurate recognition of sad faces (39,42).

Sleep deprivation induces impairments in perception of facial emotion and is associated with a diminished ability to recognize facial emotions the next morning (43–47), with impaired responses primarily found for recognition of sad facial expressions (44,45,48,49), similar to the deficits found in patients with depression (37–41). Data suggest that deficits in facial recognition in response to sleep deprivation are more reliably identified in females (47).

Insomnia, especially disturbance in sleep maintenance, alters FEP. Patients with insomnia show delays in identifying sad facial emotions and attenuated ratings of their intensity (48,49). Disturbance of sleep maintenance with increases in the amount of WASO as indexed by polysomnography, but not other measures of sleep continuity (i.e., sleep duration), correlates with delays in the recognition of sad faces and attenuated ratings of perceived emotion intensity (50). WASO also has a unique role in predicting impairments in daytime functioning (51), subsequent insomnia disorder (52), and depression in older adults (16).

## INFLAMMATION AND DEPRESSION

Activation of inflammatory signaling in the body is transduced to the brain via receptor-dependent afferent vagal mechanisms that alter neurotransmitter metabolism, brain activity, and behaviors in animals, which are broadly characterized as sickness behaviors as reviewed previously (53–55). In humans, experimentally induced inflammatory exposure induces depressive symptoms such as depressed mood and anhedonia, along with fatigue, retardation, and socioemotional withdrawal, especially in females (55). Increases in depressive symptoms are coupled with changes in the activity of brain sites that regulate reward (i.e., ventral striatum) and depressed mood (i.e., dorsal anterior cingulate cortex) (56–58).

The inflammatory biotype of depression, including that induced by insomnia, may contribute to some types of depression [as reviewed in (55,59)]. The inflammatory biotype refers to a subset of depressed patients who exhibit low-grade inflammation (i.e., C-reactive protein [CRP] >3 mg/L) (60), found in a substantial minority of patients with depression (59). These patients with depression are also likely to exhibit reduced response to antidepressant medication but favorable antidepressant response to electroconvulsive therapy or ketamine (60,61). Moreover, depression comorbidity is elevated in patients with an inflammatory disorder (14). Some prospective findings show that inflammatory markers (i.e., CRP, interleukin 6 [IL-6], and tumor necrosis factor [TNF]-α) predict depressive symptoms and depression incidence (59), although effects are attenuated by body mass index and the presence of physical disability (62). Nevertheless, exposure to inflammatory activation, whether induced experimentally (i.e., endotoxin administration), pharmacologically (i.e., interferon [IFN] doses), or naturalistically (i.e., vaccination, infection), can lead to subthreshold depressive symptoms and onset of a major depressive disorder, especially when elevated levels of inflammation persist for days and weeks or when exposure is repeated (55,63). Finally, pharmacological antagonism of endogenous inflammation can induce a reduction of depressive symptoms and clinical remission of depression in patients with an inflammatory disorder (i.e., psoriasis) and depressed patients with high levels of inflammation (64,65), although some studies have not found these benefits (66). Taken together, inflammation is viewed as a biologically plausible mechanism that contributes to depressive symptoms and, if instigated by insomnia as described below, may biologically transduce insomnia into depression.

## SLEEP AND INFLAMMATION: RECIPROCAL LINKS

Sleep and inflammatory biology demonstrate bidirectional actions. Increases in inflammation can regulate sleep in a time-, dose-, and species-dependent manner. In turn, sleep deprivation as well as insomnia lead to increases in inflammation.

### Inflammation and Regulation of Sleep

Inflammatory cytokines have a homeostatic role in the regulation of sleep. In animals, the sleep period temporally correlates with increases in brain levels of IL-1 and TNF messenger RNA and their proteins (67), with evidence that inflammatory cytokines act via cytokine-dependent receptor mechanisms to drive recovery sleep after a period of sleep loss (68). In humans, circulating levels of IL-6 and TNF increase during the nocturnal period (69), and sleep onset temporally coincides with increases in IL-6, independent of circadian processes (70). Furthermore, monocytic production of IL-6 and TNF in the evening before sleep predicts greater sleep efficiency and sleep depth (71).

In experimental models (i.e., infectious challenge), activation of inflammatory cytokines (i.e., IL-1, IL-6, TNF, type I IFN) also induces cytokine receptor–dependent increases in non–rapid eye movement (NREM) sleep and decreases in REM sleep in animals (68,72–75). Similarly, endogenous increases in inflammatory activity in response to a low dose of endotoxin induce increases in NREM sleep and slow wave sleep in humans (76), with similar effects found in rodents and rabbits (77).

In contrast to the supportive effects of endogenous levels of inflammation on sleep, pharmacological doses of IL-6 or IFN-γ have countervailing effects and induce decreases, not increases, of NREM in healthy volunteers (78) and patients with hepatitis C (79). To add further complexity, administration of the IL-1 receptor antagonist anakinra, which antagonizes endogenous levels of inflammatory cytokines, was found unexpectedly to promote increases, not decreases, in slow wave activity, as indexed by electroencephalography power spectra (80). The reason for the surprising increase in slow activity is not known but may be caused by the induction of systemic hormonal changes (i.e., prolactin) in response to IL-1 receptor antagonism, yet prolactin does not induce slow wave sleep, but rather its secretion co-occurs with increases in slow wave activity (80). In sum, neither pharmacological doses of inflammatory cytokines nor their antagonists capture the homeostatic role of endogenous cytokines in the regulation of sleep, which may be due to differences in dose and/or specificity related to these pharmacological agents.

In patients with an inflammatory condition, insomnia complaints are prevalent (3,14), raising the possibility that aberrant and chronic increases in inflammation may have pathological effects on sleep; such chronic increases in inflammatory cytokines in these patients exceed typical endogenous levels that have a homeostatic role in supporting sleep. To test the hypothesis that aberrant increases in inflammation contribute to insomnia complaints in patients with inflammatory conditions, clinical trials have examined whether pharmacological antagonism of inflammatory activity might improve sleep in these patients. Indeed, in depressed patients with elevated levels of inflammation (CRP >5 mg/L), TNF antagonism improved measures of sleep maintenance (81). Similarly, in patients with rheumatoid arthritis, TNF antagonism induced increases in slow wave sleep and improvements in sleep maintenance (82,83) and sleep quality (84–87). Furthermore, administration of an IL-6 antagonist (88) or Janus kinase inhibitor, tofacitinib (89), had salutary effects on insomnia complaints in patients with rheumatoid arthritis. One experimental study in individuals with alcohol dependence found that the TNF antagonist etanercept induced short-term (i.e., one night) decreases of REM sleep, but not NREM sleep (90). Other studies have found that disease-modifying antirheumatic drugs improve patient-reported sleep quality in patients with rheumatic arthritis (86,89,91–93). Research is needed to evaluate whether such biological therapies that target inflammation might mitigate persistent insomnia complaints in depressed patients with elevated inflammation.

### Sleep Loss and Activation of Inflammatory Biology

Sleep loss has a causal role in the activation of inflammatory signaling mechanisms. Whereas acute exposure to sleep loss (i.e., sleep deprivation) fails to induce consistent increases in morning circulating levels of inflammation such as CRP, IL-6, and TNF (94), sleep deprivation repeated over multiple nights leads to a progressive increase in systemic inflammation (i.e., CRP) (95,96). Changes in circulating level of inflammation are signaled by upstream transcriptional and cellular mechanisms, and even a single night of partial night sleep deprivation activates 2 key inflammatory transcriptional factors, nuclear factor-κB (NF-κB) and AP-1 (97,98), as well as intracellular STAT family proteins that modulate inflammatory processes (99). In turn, partial night sleep deprivation leads to increases in messenger RNA’s encoding inflammatory cytokines (97), along with increases in the cellular production of inflammatory cytokines at rest (99) and after TLR-4 stimulation of monocytes (97). Similar to sleep deprivation, disruption of sleep maintenance by experimental exposure to 2 nights of forced awakenings also induces robust activation of TLR-4–stimulated production of inflammatory cytokines, an effect that is mediated by a loss of slow wave sleep (100).

Biological heterogeneity moderates the inflammatory effects of sleep deprivation. Females show greater increases of NF-κB than males (98) and more persistent increases in the cellular production of inflammatory cytokines across the day in response to sleep loss (101). It is not known whether depressed patients with comorbid insomnia have a heightened inflammatory sensitivity to sleep deprivation, although other patients with comorbid insomnia (i.e., alcohol use disorder, rheumatoid arthritis) show enhanced spontaneous and TLR-4 stimulated monocyte production of inflammatory cytokines in response to sleep deprivation or interpersonal stress (102,103). Indeed, experimental interruption of sleep maintenance in animals drives epigenetic reprogramming, which primes immune cells toward the development of an exaggerated inflammatory response to challenge (104). Such increases in inflammation following experimental sleep deprivation may have a homeostatic role in sleep recovery, given that higher monocytic levels of inflammation before sleep onset predict greater sleep maintenance and sleep depth over the subsequent night (71).

Systemic chronic inflammation is also promoted when insomnia complaints become persistent for weeks to months (i.e., insomnia disorder). Cross-sectional meta-analytic evidence has found that self-reported sleep disturbance, as well as poor sleep quality, is associated with higher levels of CRP and IL-6 (94). Older adults with insomnia complaints show increases in TLR-4 induced production of inflammatory cytokines, along with increases in transcriptional factor activity related to immune activation (105). Furthermore, self-reported poor sleep quality prospectively predicts subsequent increases in systemic markers of inflammation (106–109), which reportedly mediate mortality risk (108,109). Importantly, a number of comorbid conditions (e.g., chronic pain, metabolic syndrome) (14) and socioeconomic and other behavioral factors might contribute to the relationship between insomnia and inflammation [as previously discussed (3,94)].

Whether insomnia-related inflammation underlies the link between insomnia and depression is not known, although individuals with chronic inflammation (i.e., men who are infected with HIV) show an exaggerated risk of depression, as noted earlier (15). Importantly, when insomnia disorder is treated with either cognitive behavioral therapy for insomnia (CBT-I) or delivery of a mind-body intervention (i.e., Tai Chi), systemic, cellular, and genomic measures of inflammation are reduced, with benefits sustained over 1 year (110–113).

Insomnia complaints in patients with depression frequently include variability of sleep duration, shorter sleep duration, and disturbance of sleep maintenance. Importantly, each of these aberrant sleep characteristics contributes to inflammation. In healthy volunteers, night to night variability in sleep duration correlates with increases in CRP levels (114). Likewise, shorter sleep duration as evaluated by either subjective or objective methods is linked to higher levels of CRP but not IL-6 (94). Larger effect sizes are found in younger and female subgroups, with exploratory results also suggesting an increased risk of inflammation in African Americans (115) and those with poor social relationships (116). Finally, disturbances of sleep maintenance with greater amounts of WASO, as assessed by sleep diaries, are associated with higher levels of activated NF-κB and higher levels of STAT1, STAT3, and STAT4 in females but not in males (117). To my knowledge, no study reported to date has examined whether insomnia complaints uniquely contribute to inflammation in patients with depression, although sleep disturbance and retardation, but not other depressive symptoms, predict a reduction in natural killer cell activity in patients with depression (118).

Chronic inflammation found in patients with insomnia and/or depressed patients with insomnia is thought to adversely affect medical outcomes (3). Moreover, sleep disturbance induces a reduced capacity for lymphoid differentiation, drives epigenetic reprogramming, and accelerates epigenetic aging of leukocytes (104,119,120). In patients with depression, it is hypothesized that insomnia may suppress hematopoietic clonal diversity and initiate changes in the epigenome, which promote epigenetic aging and sustained inflammatory output. Together, these cellular and molecular changes may lead to inflammatory comorbidities including cardiovascular disease and diabetes mellitus. The size of the effect between insomnia complaints and inflammation is similar to other risk factors known to contribute to inflammatory conditions, including older age, non-White ethnicity, female sex, higher body mass index, and sedentary behavior (94,121).

## INSOMNIA AND INFLAMMATION: A TWO-HIT MODEL OF DEPRESSION

**Figure 1.**
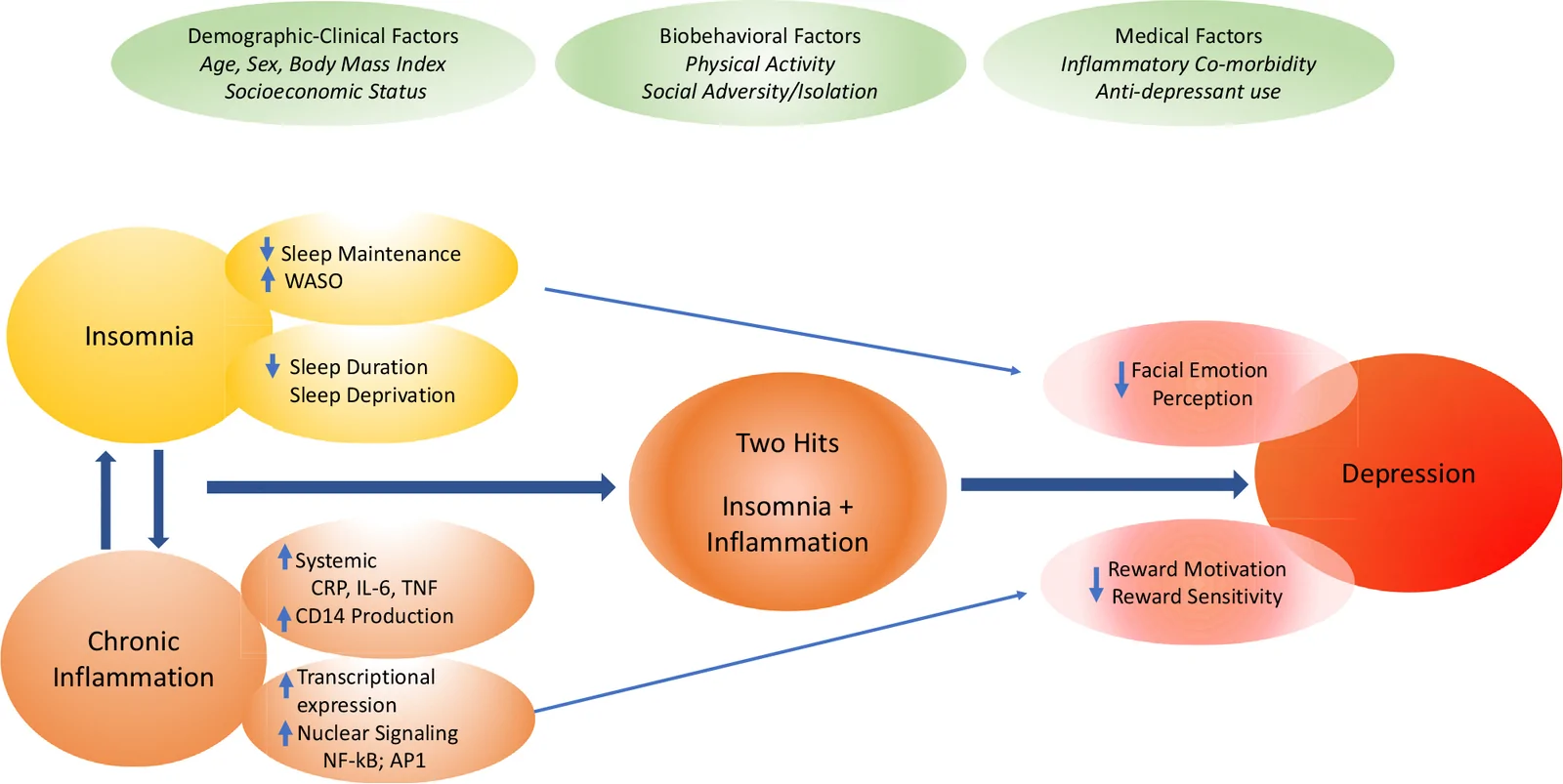
Putative two-hit model depicting the separate and combined effects of insomnia and chronic inflammation on depression. Socioenvironmental exposure such as social threat or social adversity activates inflammatory signals at low levels, similar to the endogenous increases with subclinical inflammatory disease or chronic infection. Insomnia, especially persistent difficulty maintaining sleep or short sleep duration, leads to sustained activation of these inflammatory signals, or chronic, elevated inflammation. Chronic inflammation is characterized by a shift in the transcriptional profile toward increased inflammatory activity, with increased inflammatory gene expression due to increases in transcriptional inflammatory signaling (AP-1 and nuclear factor-κB [NF-κB]). In turn, increases in downstream markers of inflammation occur, with increases in cellular inflammation (TLR-4 stimulated monocyte production of interleukin 6 [IL-6] and tumor necrosis factor [TNF]), and increase systemic inflammation (circulating concentration of C-reactive protein [CRP], IL-6, and TNF). Insomnia, especially disturbance of sleep maintenance, alters affective mechanisms including processing of reward and emotion perception and also predicts increases in depression risk. Additionally, chronic inflammation reduces reward motivation and reward sensitivity and is associated with increased risk of depression. When insomnia and chronic inflammation occur together, a two hit, delays in facial emotion perception and deficits in reward motivation and reward sensitivity are exaggerated, resulting in greater increases in depressed mood, subthreshold depressive symptoms, and possibly major depression. These pathways linking insomnia, inflammation, and depression are also influenced by biological heterogeneity (i.e., age, sex), clinical differences (i.e., body mass index, inflammatory and other medical comorbidities), and biobehavioral factors (socioeconomic and other social adversity, physical activity). This two-hit model hypothesizes that insomnia and inflammation conspire to alter affective mechanisms and increase the likelihood of major depressive disorder. WASO, wake after sleep onset.

Given that insomnia as well as sleep deprivation is associated with increases in inflammation and that inflammation can elicit depressive symptoms, the inflammatory biotype may be a salient factor by which insomnia is converted into affective risk and depression (Figure 1) (122). Indeed, in patients with a chronic inflammatory disorder including asthma, cardiovascular disease, or rheumatoid arthritis, insomnia complaints and depression are often comorbid (14). Furthermore, experimental sleep deprivation induces increases in depressed mood, which are exaggerated in patients with an inflammatory disorder (17). In addition, experimental sleep deprivation induces increases in cellular inflammation (i.e., TLR-4 monocytic production of inflammatory cytokines), which is greater in adults compared with older adults (>60 years) and in females compared with males (101). These findings have implications for understanding the greater risk of depression in younger adults, especially younger females compared with males, and older adults (123). Finally, a laboratory-based study suggests that inflammation heightens the association between poor sleep maintenance and depressed mood (124). Disturbance of sleep maintenance (i.e., WASO), as objectively assessed by polysomnography, is associated with increases in depressed mood the following morning, and this depression response is moderated by morning levels of inflammation (i.e., IFN-γ); those with the greater amounts of WASO along with higher levels of IFN-γ show a greater depression response (124). Together, these data support the notion of a two-hit model of depression, in which insomnia and inflammation jointly contribute to depressed mood (122).

Insomnia is also associated with alterations in affective mechanisms, including reward processes and facial emotion recognition, as noted earlier, and such dysregulation is heightened when inflammation is elevated. In older adults with insomnia, higher levels of CRP in males, but not females, magnify the reduction in reward motivation (28). Furthermore, the association between poor sleep maintenance and emotional perception of sad faces is moderated by inflammatory cytokines; the association between WASO and deficits in emotion recognition of sad faces, or rating of intensity of sad emotion, is greater among those older adults who had higher levels TNF or IL-6, respectively (50). Conversely, lower levels of TNF and/or IL-6 might buffer the adverse effects of disturbance of sleep maintenance on emotion regulation.

Inflammation shows dynamic variability, due in part to many contributing factors, including specific diseases (i.e., infections) and psychosocial factors (i.e., interpersonal stress) (59). Given that inflammatory exposure can lead to depressive symptoms and that depression responses after a night of sleep disturbance are heightened in those with higher levels of inflammation, we have hypothesized that inflammatory exposure may induce greater increases in depressed mood and depressive symptoms in individuals with insomnia compared with those without insomnia. Experimental substantiation of this hypothesis would provide support for a mechanistic link between insomnia, inflammation, and depression, by testing whether inflammatory exposure causes an exaggerated increase in depressive symptoms in individuals with insomnia.

Indeed, in a randomized controlled trial of 160 older adults, we recently reported that experimental administration of the inflammatory challenge, low-dose endotoxin versus placebo, induced robust increases in depressed mood and clinician-rated depressive symptoms (125). Furthermore, among those with insomnia, endotoxin induced exaggerated increases in depressed mood and depressive symptoms, which persisted for more than 6 hours, compared with small and transient increases in older adults without insomnia (125). Importantly, among those with insomnia, increases in depressive symptoms in response to endotoxin were clinically important differences with peaks in the mild depression range (125); subthreshold depressive symptoms predict diagnostic depression in older adults (126). Moreover, these findings have further clinical implications, given that naturalistic inflammatory exposures (i.e., infection, flare of an inflammatory disorder) yield greater increases and more persistent inflammation, possibly inducing enduring subthreshold depressive symptoms and incident depression (14,59). In short, those with insomnia should be vigilantly monitored for depression during times of inflammatory exposure and might also benefit from treatments that target inflammation to prevent and treat depression.

Whether these findings extend to other at-risk populations is not known, although previous research has found that greater inflammatory reactivity in response to an acute interpersonal stressor is longitudinally correlated with greater acute increases in depressive response (59,127) and depressive symptoms over the following year in healthy adults without insomnia (127).

## INSOMNIA AND DEPRESSION TREATMENT OUTCOMES: POTENTIAL ROLE OF INFLAMMATION

In nondepressed patients with insomnia complaints, it is well known that insomnia treatments, such as CBT-I, yield robust and more durable rates of insomnia response and remission than sedative-hypnotic pharmacotherapy (128). Additionally, community accessible mind-body interventions such as Tai Chi and mindful meditation have been found to yield insomnia treatment responses that are noninferior to CBT-I (129,130). Importantly, insomnia remission temporally coincides with improvements in depressed mood and subsyndromal depressive symptoms (129–131).

In depressed patients who are comorbid with insomnia complaints, the severity of insomnia is associated with less favorable response to depression treatments (9). Whether measured subjectively or objectively using actigraphy or polysomnography, insomnia symptoms are related to greater rates of attrition, poorer rates of remission, and increased likelihood of depression recurrence in response to pharmacological, behavioral, or psychological depression treatments [as reviewed in (9)]. Furthermore, self-reported insomnia complaints lead to a blunted response to depression treatment (132). Taken together, these observational findings raise the possibility that treatment of insomnia, coupled with depression therapies, might result in a more robust and/or sustained remission of depression.

Two small studies targeted residual insomnia symptoms and found that the addition of CBT-I to standard treatment induced greater improvements in both insomnia and depression symptoms and rates of remission (133,134). Other studies have treated depression and comorbid insomnia disorder with concomitant CBT-I and antidepressant medications compared with medications only, but findings have been mixed. Although CBT-I effectively reduced insomnia symptoms in patients with depression, CBT-I was not found to improve rates of depression remission or symptom reduction in response to the antidepressant medication escitalopram (9,133–136). However, there is some suggestion that early improvements in insomnia symptoms may predict subsequent depression remission over a 6-month follow-up (136).

Given that only approximately one-third of patients with depression achieve remission in response to pharmacotherapies, leading to a persistent burden of depression, effective prevention of depression is also urgently needed. In a recent meta-analysis, a variety of psychological interventions (i.e., interpersonal psychotherapy, CBT) were found to be effective in reducing depression incidence and depressive symptom severity with moderate efficacy (137). However, the vast majority of studies have enrolled adults already experiencing subthreshold depressive symptoms. Furthermore, most previous trials did not administer targeted approaches (i.e., insomnia treatment), which are needed to improve treatment efficacy (i.e., reduce the number needed to treat).

To evaluate whether targeting insomnia with CBT-I could prevent the incidence of major depressive disorder in older adults with insomnia, a selective depression prevention trial was recently reported (138). In 291 nondepressed older adults with insomnia, CBT-I compared with an active comparator control, sleep education therapy (SET), was administered for 2 months with repeated assessment of DSM-5 recurrent and incident major depressive disorder over a 3-year follow-up. Compared with SET, CBT-I reduced the likelihood of incident and recurrent major depressive disorder by nearly 50%. Among those older adults who received CBT-I and had sustained (i.e., 3 years) remission of insomnia, there was an 83% reduction in the likelihood of depression (138). Treatment of insomnia has an overall benefit in the prevention of incident and recurrent major depression in nondepressed older adults with insomnia disorder. Separately, 2 previous studies enrolled insomnia patients with subsyndromal depressive symptoms and used digitally based insomnia treatment programs. However, findings were limited to the reduction of depressive symptoms in this indicated sample, without evidence of prevention of incident depression, possibly due to poor treatment adherence and short-term follow-up (139–141).

To advance the efficacy of interventions that target insomnia to treat and/or prevent depression, subgroup differences need to be considered to improve engagement, retention, and effectiveness (142–144). Using individual participant data from 30 randomized controlled trials with 7201 participants, psychological interventions to prevent depression incidence showed stronger effects among those who had not previously received psychotherapy and had more severe depressive symptoms at enrollment (137); neither age nor sex was identified as a moderator. Whether these findings generalize to trials that target insomnia is not known; almost no studies have considered the mechanisms that contribute to the efficacy of insomnia treatment on depression outcomes. One trial found that circadian preference moderated improvements in depressive symptoms in response to antidepressant medication plus CBT-I (145). The inflammatory biotype may also alter the effectiveness of insomnia therapies on depression outcomes. As noted earlier, remission of insomnia reduces inflammation in nondepressed patients with insomnia (111–113), but it is not known whether such decreases in inflammation mediate or moderate subsequent depression outcomes. Further analyses, using Bayesian and other causal statistical frameworks, are needed to infer potential causal relationships. Indeed, such approaches are underway to examine whether CBT-I and sustained remission of insomnia reduce inflammation and mediate the prevention of depression in older adults with insomnia.

## CONCLUSIONS

Environmental and genomic factors influence sleep, as well as inflammation, and further research is needed to understand how these individual, within-person factors initiate and perpetuate a misalignment of sleep and immunological homeostatic mechanisms. Insight into how these distinct aspects lead to changes in sleep continuity, sleep architecture, and immunological signatures will inform the development of personalized therapies to target these interrelated mechanisms that contribute to dysregulation of reward and emotion processes, depressive symptoms, and incident depression.
